# Anti-inflammatory effects of fish oil in ovaries of laying hens target prostaglandin pathways

**DOI:** 10.1186/1476-511X-12-152

**Published:** 2013-10-24

**Authors:** Erfan Eilati, Carolynn C Small, Stacey R McGee, Nawneet K Kurrey, Dale Buchanan Hales

**Affiliations:** 1 Department of Physiology, Southern Illinois University Carbondale, School of Medicine, Life Science II, Room 250 (M/C 6512), 1125 Lincoln Drive, Carbondale, IL 62901, USA

**Keywords:** Fish oil, Laying hen, Omega-3 fatty acids, Inflammation, Cyclooxygenase, Prostaglandin E_2_

## Abstract

**Background:**

An effective way to control cancer is by prevention. Ovarian cancer is the most lethal gynecological malignancy. Progress in the treatment and prevention of ovarian cancer has been hampered due to the lack of an appropriate animal model and absence of effective chemo-prevention strategies. The domestic hens spontaneously develop ovarian adenocarcinomas that share similar histological appearance and symptoms such as ascites and metastasis with humans. There is a link between chronic inflammation and cancer. Prostaglandin E_2_ (PGE_2_) is the most pro-inflammatory ecoisanoid and one of the downstream products of two isoforms of cyclooxygenase (COX) enzymes: COX-1 and COX-2. PGE_2_ exerts its effects on target cells by coupling to four subtypes of receptors which have been classified as EP1-4. Fish oil is a source of omega-3 fatty acids (OM-3FAs) which may be effective in prevention of ovarian cancer. Our objective was to assess the potential impact of fish oil on expression of COX enzymes, PGE_2_ concentration, apoptosis and proliferation in ovaries of laying hens.

**Methods:**

48 white Leghorn hens were fed 50, 100, 175, 375 and 700 mg/kg fish oil for 21 days. The OM3-FAs and omega-6 fatty acids contents of egg yolks were determined by Gas Chromatography. Proliferation, apoptosis, COX-1, COX-2 and prostaglandin receptor subtype 4 (EP4) protein and mRNA expression and PGE_2_ concentration in ovaries were measured by PCNA, TUNEL, Western blot, quantitative real-time qPCR and ELISA, respectively.

**Results:**

Consumption of fish oil increased the incorporation of OM-3FAs into yolks and decreased both COX-1 and COX-2 protein and mRNA expression. In correlation with COXs down-regulation, fish oil significantly reduced the concentrations of PGE_2_ in ovaries. EP4 protein and mRNA expression in ovaries of hens was not affected by fish oil treatment. A lower dose of fish oil increased the egg laying frequency. 175 and 700 mg/kg fish oil reduced proliferation and 700 mg/kg increased apoptosis in hen ovaries.

**Conclusions:**

Our findings suggest that the lower doses of fish oil reduce inflammatory PG and may be an effective approach in preventing ovarian carcinogenesis. These findings may provide the basis for clinical trials utilizing fish oil as a dietary intervention targeting prostaglandin biosynthesis for the prevention and treatment of ovarian cancer.

## Introduction

The best approach for reducing the impact of ovarian cancer is by prevention. Fish oil supplementation as a source of omega-3 fatty acids (OM-3FAs) might be effective in prevention and amelioration of the severity of the ovarian cancer by targeting inflammatory prostaglandin pathways.

Cyclooxygenase (COX) is the rate limiting enzyme in catalyzing the conversion of arachidonic acid (AA) to prostaglandins (PGs). Two isoforms of cyclooxygenase have been identified, COX-1 and COX-2. Although both COX isoforms have similar structure and function, they are encoded by different genes (PTGS1 and PTGS2) and show distinct expression patterns. COX-1 is expressed in most cells and tissues and remains constant under most physiologic conditions to play a housekeeping role whereas the COX-2 form is inducible and usually only expressed in response to various inflammatory stimuli [1]. COX enzymes may be involved in both tumor establishment [2] and maintenance of existing tumors [3]. Up-regulation of COX-2 has been reported in many malignancies including breast [4], lung [4] and ovarian cancer [5]. However, we and others have shown that COX-1 is over-expressed in ovarian cancer [3,6,7].

PGs are biologically active lipids that are associated with inflammation, fever, pain and tissue injury [8]. High concentrations of PGE_2_ are found in different human cancers including colon, lung, breast, and head and neck cancers [9]. Prostaglandins and their metabolites enhance the cell proliferation, inhibit apoptosis and stimulate cell adhesion. PGE_2_ exerts its autocrine/paracrine effects on target cells by coupling to four subtypes of G-protein coupled receptors, which have been classified as EP1-4 [10]. EP4 is over-expressed in many cancers such as castration-resistant prostate cancer [11] and colorectal cancer [12] and EP4 antagonists inhibit breast cancer metastasis in mice [13].

Ovarian cancer is the fifth leading cause of cancer death among women and the most lethal gynecological malignancy. Research into the discovery of early detection markers and therapeutics continues, but there are still no effective treatments for ovarian cancer today [14]. It is clear that poor diet is a well established risk factor for cancer. In fact, the American Institute for Cancer Research estimates that 30–40 percent of all cancers can be prevented by appropriate diets, physical activity and maintenance of appropriate body weight [15]. Omega-6 fatty acids (OM-6FAs) and OM-3FAs are essential poly unsaturated fats that must be obtained from diet. OM-6FAs can be found in red meat, chicken and vegetable seed oil such as sunflower seed oil. OM-3FAs can be found in flaxseed and in oily cold water fish such as salmon and tuna. The main OM-6FA, Linoleic Acid (LA), is a direct precursor of the Arachidonic Acid (AA). The OM-3FAs include alpha-linolenic acid (ALA), eicosapentaenoic acid (EPA), and docosahexaenoic acid (DHA). In early human diets, the ratio of OM-6FAs to OM-3FAs was approximately equal; however, with the evolution of the modern western diets, the ratio favored the OM-6FAs that have been attributed to the increased risk of cardiovascular disease and cancer [16]. In contrast, changing the ratio in favor of OM-3FAs has been shown to have growth suppressive effects on cancerous cells in animal studies [17]. Epidemiological evidence and preclinical data suggest that consumption of OM-3FAs might be associated with prevention of many cancers in humans likely due to reduction in inflammatory prostaglandins. However, the mechanism(s) by which the OM-3FAs in dietary fish oil, EPA and DHA, have anti-neoplastic activity remains unclear [18].

Hens spontaneously develop ovarian adenocarcinomas that are similar to human disease by histopathology [19], expression molecular markers [20-22] and presentation of symptoms such as perfuse ascites fluid and peritoneal metastatic dissemination [19,23].

Previously we have shown that increased expression of both COX enzymes and concentration of PGE_2_ in hen ovaries correspond to the increased incidence of ovarian cancer indicating their importance in early stages of ovarian cancer [24]. Moreover, we demonstrated that the long-term consumption of flaxseed (richest plant source of OM-3FAs) reduced the incidence and severity of ovarian cancer in hens which correlated with reduced COX-2 and PGE_2_[25]. Thus, targeting COX expression and prostaglandin biosynthesis by dietary intervention using fish oil starting at young ages might be an effective approach to prevent or suppress ovarian cancer later in life. The objective of this study was to assess the effect of fish oil on COX-1 and COX-2 enzyme expression and PGE_2_ concentrations in normal ovaries of laying hens.

## Material and methods

### Animal care

48 single comb white Leghorn hens, aged one year were used. Hens were maintained two per cage and were provided with feed and water ad libitum and exposed to a photoperiod of 17 h light: 7 h dark, with lights on at 05:00 h and lights off at 22:00 h. Animal management and procedures were reviewed and approved by the Institutional Animal Care and Use Committees at the University of Illinois at Urbana-Champaign and Southern Illinois University at Carbondale.

### Experimental design

This study was performed in two parts. For the first part, 24 hens were randomly divided into four groups (n=6). Three groups were fed 175, 375 and 700 mg/kg/day of fish oil filled into the gelatin capsules and one group was fed empty gelatin capsules. For the second part, 24 hens were randomly divided into four groups (n=6) and three groups were fed 50, 100 and 175 mg/kg/day of fish oil and the empty gelatin capsules were given to the control group. Eggs were collected daily to determine ovulation rate which is reported as number of laid eggs per hen per week. To ensure that the OM-3FAs from fish oil were incorporated into the ovaries, four eggs from all groups were collected on days 0, 7, 14 and 21 of the study and measured for total OM-3FAs and OM-6FAs contents by gas chromatography. If enough eggs were not laid on a given day, the last laid eggs from previous day were used. EPA and DHA contents of fish oil capsules (500 mg omega-3 Salmon oil, Puritan’s Pride, Oakdale, NY, USA) were measured and the manufactures reported amounts confirmed. No overt toxicity was observed at any dose.

### Collection of tissue

After 21 days, all hens were euthanized using CO_2_ asphyxation and necropsied. Ovaries were removed from hens and small yellow follicles (6–8 mm) and pre-ovulatory follicles (9–35 mm) were removed. The ovaries were dissected into several pieces. The first portion was frozen in liquid nitrogen and later stored at −80°C; the second portion was put into RNAlater solution and stored at 4°C before processing; the third and fourth portions were used for histological analysis and fixed in NBF fixative solution.

### Gas chromatography

Measuring OM-3FA in egg yolks is a non-invasive approach in the poultry industry to measure total body OM-3FA incorporation [26,27]. The yolk was separated from the rest of the egg. A solution containing 12.5 ug/ml C17:0 standard in methanol was then added to the egg yolks. Lipid extraction was performed using HPLC grade chloroform, water and HPLC grade hexane. The lipids were then dried under a stream of nitrogen and methylated using the Instant Metanolic HCl kit (Alltech, Lexington, KY, USA). After methylation, the lipids were extracted twice with hexane. Once dried under nitrogen, the lipids were re-dissolved in hexane and injected into the Shimadzu 2010 gas chromatograph equipped with a flame ionization detector. Helium was used as the carrier gas and nitrogen as the make up gas with an Omega-wax column. The injection volume was 1.0 μL, helium was used as the carrier gas (30 cm/sec, 205°C), and Helium as the make-up gas with an Omegawax (250, 30 m × 0.25 mm I.D., 0.25 *μ*m film) column. The injector temperature was 250°C. A split injection technique (100:1) was used, and the temperature program was as follows: 175°C held for 8 min, increased to 200°C at 4.5°C/min, and held at 200°C for 8 min, then increased to 230°C at 6°C/min, and held at 230°C for 14 min. Individual FAME were identified by reference to external standards (PUFA-3, and C17:0 Sigma-Adrich). Protein levels were determined by Pierce BCA Protein Assay. Fatty acids were quantified by integration of area under peak after normalization to C17:0 standards and to ug/ug protein.

### RNA extraction and analysis

Total RNA was extracted from ovarian tissue using Trizol reagent. Quantification was performed by determination of absorbance at A260, and qualified by Experion RNA StdSens Analysis. All RNA samples used in this study had a 260:280 ratio range between 2 and 2.10, and had three bands: 5S, 18S and 28S for electrophoresis results. RNA samples were then treated with RQ1 RNase-free DNase prior to reverse transcription reaction. cDNA was synthesized from DNase treated RNA with the high capacity cDNA archive kit.

### Quantitative real-time PCR

The chicken-specific primers and plasmid standards used for each gene were designed. Primer sequences are shown in Table 1. qRT-PCR was conducted by amplifying cDNA with Eva-Green on CFX384 Real-Time System and analyzed with Bio-Rad CFX Manager software. Control reactions lacking template were run for each gene. Reactions were 10 μl in total volume and 200 nM of each primer. The amplification conditions were as follows: 50°C 5 S, 95°C 10 min, 40 cycles for 95°C 15 S, 60°C 1 min.

**Table 1:** Primer sequences

		
COX-1	Forward	5′TCAGGTGGTTCTGGGACATCA 3′
	Reverse	5′ TGTAGCCGTACTGGGAGTTGAA 3′
COX-2	Forward	5′ CTGCTCCCTCCCATGTCAGA 3′
	Reverse	5′ CACGTGAAGAATTCCGGTGTT 3′
EP4	Forward	5′ GGTGTTTCATAGACTGGCGA 3′
	Reverse	5′ GCAGATCACCGTAACCATGA 3′
SDHA	Forward	5′ CAGGGATGTAGTGTCTGCT 3′
	Reverse	5′ GGGAATAGGCTCCTTAGTG 3′
TBP	Forward	5′ CGTCAGGGAAATAGGCA 3′
	Reverse	5′ GACTGGCAGCAAGGAAG 3′
RPL4	Forward	5′ TTATGCCATCTGTTCTGC 3′
	Reverse	5′ GCGATTCCTCATCTTACCCT 3′

### Western blot

The proteins were extracted from snap frozen ovarian tissue samples and the western blot was performed to detect COX-1 and COX-2 as described previously [24].

### Apoptosis assay

The presence of apoptotic cells were detected with the TUNEL Apoptosis Detection biotin-labeled POD Kit according to manufacturer’s protocol (GenScript, Piscataway, NJ, USA). Briefly, after deparaffinization of 5 μm PPFE sections, they were washed with phosphate buffered saline (PBS) and incubated with 0.02 mg/ml proteinase K at 37 C for 20 min. Cells were blocked with 3% hydrogen peroxide in methanol for 10 min at RT. Sections were incubated with a TUNEL labeling mixture of terminal deoxytransferase (TdT) and biotinylated dUTP at 37 C for 1 hr, followed by two washes in PBS for 5 min each. Apoptotic cells were visualized with a streptavidin conjugated antibody (Life Technologies, Carlsbad, CA, USA; Alexa Fluor 488) at RT for 30 min. After being washed with PBS, the sections were coverslipped using Fluormount G with Dapi mounting media. For control staining, the positive control section was treated for 15 min at 37 C with DNase I (25 U/ul) before incubation with TUNEL labeling mix while the negative control section was incubated in the TUNEL labeling mix without TdT.

### Proliferation assay

Proliferating Cell Nuclear Antigen (PCNA) was used to assess cell proliferation. Normal and cancerous ovaries were fixed in 10% normal buffered formalin and paraffin embedded blocks (PPFE) were sectioned at 5 μm. Sections were deparaffinized in xylene and rehydrated in reducing concentrations of ethanol. Antigen retrieval was performed in a pressure cooker using unmasking solution (Sigma) for 20 min, depressurized for 10 min, and then allowed to cool to RT for 20 min changing ¼ of the water every 5 min. The sections were treated with 3% hydrogen peroxide in methanol for 30 min at room temperature (RT) to block endogenouse peroxidase activity and rinsed in Tris Buffered Saline with 0.5% tween (TTBS). Non specific binding was blocked using 2.5% normal horse serum for 1 hr at RT then incubation with the mouse monoclonal anti-PCNA antibody (Abcam, Cambridge, MA, USA) for 16–18 hr at 4 C. Slides were washed with TTBS and incubated with donkey anti mouse Alexa Fluor 488 secondary antibody (Jackson Immuno Research, West Grove, PA, USA). for 1 hr at RT. Coverslips were applied using Fluoromount G with Dapi mounting media (Southern Biotech, Birmingham, Alabama, USA). Negative controls were incubated without the primary antibody and showed no staining.

### TUNEL and PCNA analysis

Sections stained with either terminal deoxynucleotidyl transferase dUTP nick end labeling (TUNEL) or the proliferating cell nuclear antigen (PCNA) assay were analyzed for positive stained cells in relation to the total number of cells present. At least 3 animals from each treatment group were serial sectioned. Sections from three areas of the ovary were selected with at least 100 μm between sections. Pictures of four different areas of each section were taken at 40×. Image J software was utilized to randomly mark 10 regions of interest (ROI) in each area of each section. The total number of cells was determined in these ROIs by nuclear Dapi staining. Percent of proliferation or apoptosis was determined by the average positive stained cells divided by the average total number of cells multiplied by 100.

### PGE_2_ EIA

The amount of PGE_2_ in ovarian tissues from hens in all groups was measured using a specific enzyme immunoassay as shown previously [24].

### Statistical analysis

All experiments were performed in duplicate and differences in data from groups were analyzed with GraphPad InStat by using One-way ANOVA with Student–Newman–Keuls. A value of P<0.05 was considered significant whereas a value of P<0.01 was considered highly significant.

## Results

### Amounts of OM-3FAs and OM-6FAs in egg yolks

**Figure 1:**
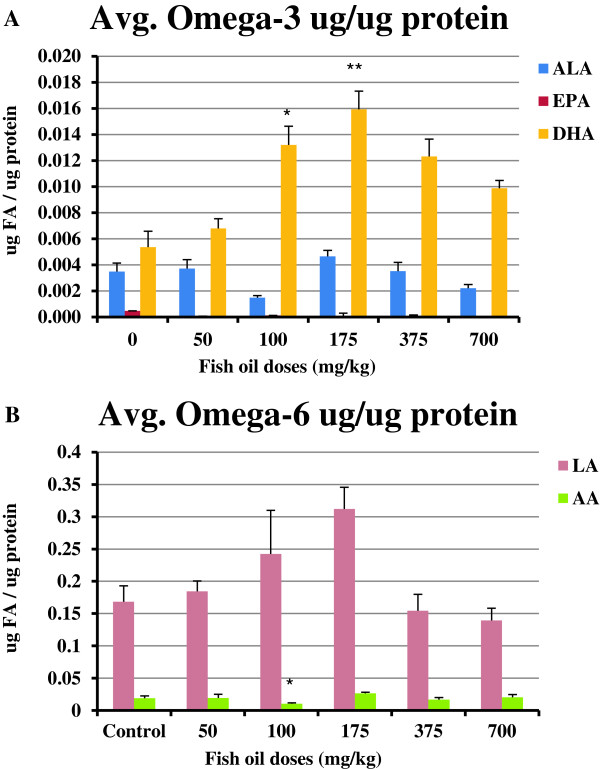
**The amounts of OM-3FAs and OM-6FAs in egg yolks.** Four egg yolks were collected from each group (n=4). **(A)** More DHA was detected in yolks collected from hens treated with 100 mg/kg (P<0.05) and 175 mg/kg (P<0.01) fish oil compared to control hens. EPA was not detectable in most of the treated groups or it was extremely low. **(B)** The yolks collected from hens treated with 100 mg/kg fish oil had significantly lower amounts of AA compared to yolks collected from control hens (P<0.05). *, P<0.05; **, P<0.01. Bars indicate standard error.

The results indicated that there were significantly higher amounts of DHA in yolks collected from hens fed 100 mg/kg (P<0.05) and 175 mg/kg (P<0.01) fish oil compared to amounts of DHA in yolks collected from control hens (Figure 1A). EPA was not detectable in most groups or it was extremely low. The yolks collected from hens fed 100 mg/kg fish oil had significantly lower amounts of AA compared to yolks collected from control hens (Figure 1B; P<0.05).

### Effect of fish oil on COX-1 mRNA and protein expression in ovaries

**Figure 2:**
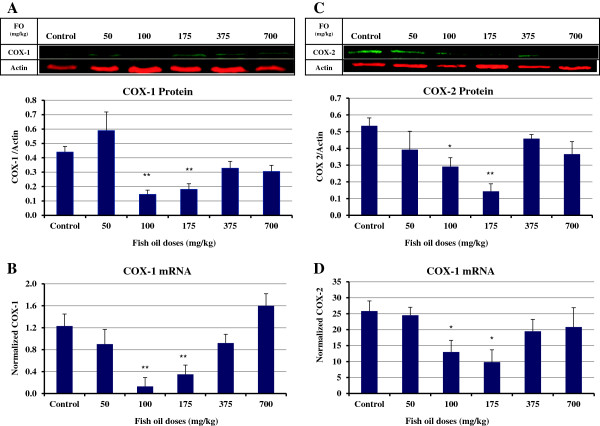
**COX-1 and COX-2 expression in ovaries of control and fish oil-fed hens. (A & B)** The hens treated with 100 mg/kg (n=6) and 175 mg/kg (n=8) fish oil had significantly lower COX-1 protein and mRNA expression compared to ovaries of control hens (n=8; P<0.01). **(C & D)** A significant reduction in expression of COX-2 protein and mRNA was seen in ovaries of hens treated with 100 mg/kg fish oil (n=6) compared to control hens (n=8; P<0.05). The ovaries of hens fed 175 mg/kg fish oil (n=8) had the lowest COX-2 protein expression among groups which was significantly lower than control hens (n=8; P<0.01). There was a significant decrease in COX-2 mRNA expression in ovaries of chickens treated with 175 mg/kg (n=8) fish oil in comparison to control hens (n=8; P<0.05). *, P<0.05; **, P<0.01. Bars indicate standard error.

Comparing the COX-1 protein expression in ovaries of hens revealed that the groups of hens that were fed 100 and 175 mg/kg fish oil had significantly lower COX-1 protein expression compared to ovaries of control hens (P<0.01; Figure 2A). Similar to the protein expression, a significant reduction in expression of COX-1 mRNA was observed in ovaries of the hens fed 100 and 175 mg/kg fish oil compared to control hens (P<0.01; Figure 2B).

### Effect of fish oil on mRNA and protein COX-2 expression in ovaries

A significant decrease in expression of COX-2 protein and mRNA was seen in ovaries of hens fed 100 mg/kg fish oil compared to control hens (P<0.05; Figure 2C & D). The ovaries of hens fed 175 mg/kg fish oil had the lowest COX-2 protein expression among groups which was significantly lower than control hens (P<0.01). There was a significant decrease in COX-2 mRNA expression in ovaries of chickens fed 175 mg/kg fish oil in comparison to control hens (P<0.05; Figure 2D).

### Fish oil consumption decreased PGE_2_ concentrations in ovaries

**Figure 3:**
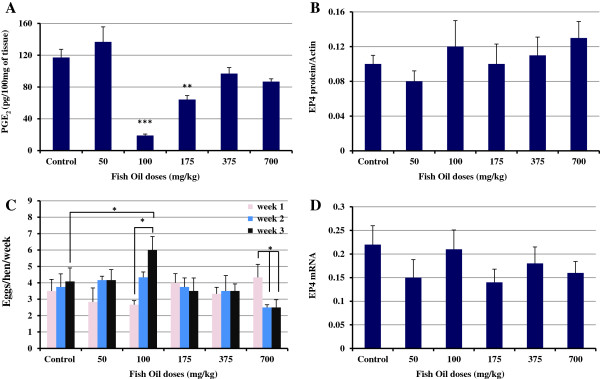
**PGE**_**2 **_**concentration in ovaries. (A)** The hens treated with 100 mg/kg (n=6; P<0.001) and 175 mg/kg fish oil (n=8; P<0.01) had lower concentrations of PGE_2_ in the ovaries compared to ovaries of control hens (n=8). **(B & D)** There were no significant differences in expression of EP4 among groups (P>0.05). **(C)** After 3 weeks of fish oil supplementation, the egg laying frequency of hens that were treated with 100 mg/kg fish oil was significantly increased compared to the ovulation rate of the same group on first week and ovulation rate of control group on third week (P<0.05). The ovulation rate in 700 mg/kg fish oil-treated hens was significantly decreased in weeks two and three compared to week one (P<0.05). *, P<0.05; **, P<0.01; ***, P<0.001. Bars indicate standard error.

In parallel with the results that we obtained for expression of COX enzymes, significantly lower concentrations of PGE_2_ were detected in ovaries of hens fed 100 mg/kg fish oil compared to ovaries of control hens (P<0.001; Figure 3A). Furthermore, feeding the hens with 175 mg/kg fish oil significantly reduced the PGE_2_ concentrations in ovaries compared to ovaries of control hens (P<0.01).

### Effect of fish oil on EP4 mRNA and protein expression in ovaries

There were no significant differences in expression of EP4 among groups indicating that fish oil does not affect the expression of EP4 protein and mRNA in ovaries of hens (Figure 3B & D).

### Effect of fish oil on Egg laying frequency

At the end of first week of the study, the ovulation rate (egg laying frequency) of all fish oil-fed hens was compared to control group, no significant differences were seen. Fish oil did not have any effect on ovulation rate of hens compared to control hens in second week (P>0.05; Figure 3C). After being on fish oil for 3 weeks, the egg laying frequency of hens that were fed 100 mg/kg fish oil was significantly increased compared to the ovulation rate of the same group on first week and ovulation rate of control group on third week (P<0.05). The ovulation rate in 700 mg/kg fish oil-fed hens was significantly decreased in weeks two and three compared to week one (P<0.05).

### Effect of fish oil on apoptosis and proliferation

**Figure 4:**
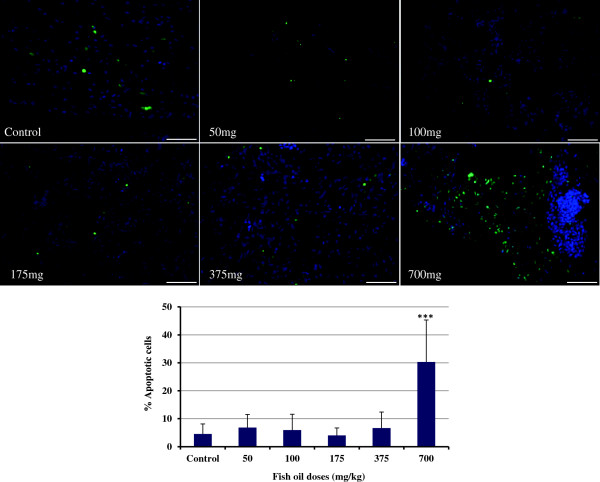
**TUNEL staining of the ovaries.** Supplementation with 700 mg/kg fish oil increased the number of apoptosis in the hen ovaries (P<0.001). Bars indicate standard error.

**Figure 5:**
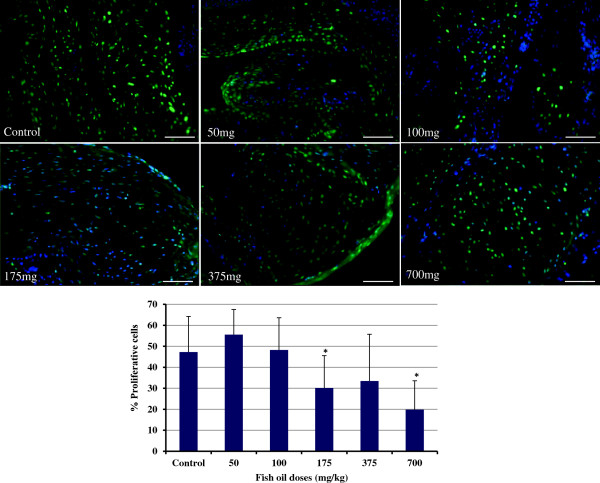
**PCNA staining of the ovaries.** The number of proliferative cells in ovary sections in hens fed 175 and 700 mg/kg fish oil was substantially lower than controls (P<0.05). Bars indicate standard error.

As shown in Figure 4, 700 mg/kg fish oil increased the rate of apoptosis in ovaries, whereas the other doses failed to show such an effect (P<0.001). The assessment of cell proliferation by PCNA staining is an indicator of proliferative growth. We observed that the population of PCNA-positive cells in ovary sections was substantially lower than controls when the hen’s diet were supplemented with 175 and 700 mg/kg fish oil (P<0.05; Figure 5).

## Discussion

Here, we report that the consumption of fish oil significantly increases the DHA incorporation into egg yolks, decreases the AA contents of yolks, and reduces both COX-1 and COX-2 protein and mRNA expression in hen ovaries. Moreover, in correlation with down-regulation of COX enzymes, fish oil significantly reduces the concentrations of PGE_2_ in ovaries. EP4 protein and mRNA expression in ovaries of hens was not affected by fish oil treatment. A lower dose of fish oil increased the egg laying frequency. 175 and 700 mg/kg fish oil reduced proliferation and 700 mg/kg increased apoptosis in hen ovaries. The efficacy of fish oil in reducing COX expression and PGE_2_ concentration was in “U-shaped curve” manner meaning that the increasing doses of fish oil correlate with reduction of COX and PGE_2_ until an optimal dose is reached beyond which further dose increases result in less effect. This finding was correlated to an “inverted U-shaped curve” of DHA amounts found in egg yolks. The concept of “U-shaped curve” refers to a nonlinear relationship between two variables, in this case fish oil dose and prostaglandin production (or COX expression). The nonlinear response, typical of many biological systems, indicates that in the optimum dose-range (100–175 mg/kg) prostaglandin production was maximally inhibited. At higher doses of fish oil the hens react in an adverse way and do not tolerate the treatment.

Incorporation of OM-3FAs into egg yolks of fish oil-fed hens provides a non-invasive measure of the incorporation of OM-3FAs into ovaries of the hens. EPA is normally found in much lesser abundance than DHA in tissues and no specific physiological response is directly attributable to EPA [4,28]. Equal amounts of DHA and EPA were detected in fish oil (data not shown) but our data showed that EPA was not detectable or was extremely low in egg yolks collected from all groups. It is likely due to its conversion to DHA by Δ^6^-desaturase. DHA is the most prominent OM-3FA in the egg yolk [29] and we detected higher amounts of it compared to ALA and EPA in yolks. DHA contents of egg yolks collected from hens fed 100 and 175 mg/kg fish oil were significantly higher compared to controls. We did not detect higher amounts of DHA in egg yolks of hens fed higher doses of fish oil. Broughton *et al.* has reported that low EPA/DHA ingestion resulted in nearly as much total OM-3FA incorporation into ovarian tissue as high EPA/DHA ingestion, indicating that ovarian tissue OM-3FA incorporation may be saturable [30]. This may explain why when the fish oil was fed in higher than a certain dose, the amounts of OM-3FAs in the egg was not altered to a greater extent. DHA contents of yolks of the hens fed 700 mg/kg were lower than DHA content of yolks collected from lower doses. Feeding the hens with 700 mg/kg fish oil for one week resulted in malabsorption of lipids and a diarrhetic response which may explain the lower DHA content of yolks. In general, our data indicated that fish oil increases the incorporation of OM3-FAs into the eggs which was in agreement with previous studies [31,32].

The amounts of AA in yolks collected from 100 mg/kg fish oil-fed hens were lower than the amounts of AA in yolks collected from the control group. It might be due to replacement of AA by DHA in membranes of ovarian cells as suggested by Schreiner *et al.* who reported that after treating hens with OM-3FAs, there was an almost complete exchange of AA and DHA in the *sn*-2 position of phosphatidylcholine and phosphatidylethanolamine in the egg yolk [29]. Meluzzi *et al.* reported that the feeding with fish oil caused a dramatic increase in all OM-3FAs of the yolk and caused an appreciable decrease in AA [32]. They proposed that this phenomenon is probably due to the greater utilization of Δ^6^-desaturase in the OM-3FA pathway with respect to the OM-6FA pathway, as this enzyme acts in both pathways [32]. Moreover, they noted that a lower dose of fish oil decreased AA in yolks more than a higher dose. High concentrations of dietary OM-3FAs reduce the activity of the enzyme in the OM-6FAs pathway and the conversion of linoleic acid into AA which could be important for human health, as this acid is a precursor of pro-inflammatory eicosanoids namely PGE_2_[33].

The molecular basis for the health benefits of dietary fish oil is likely due to incorporation of EPA and DHA into the membrane [4]. The fatty acid composition of phospholipids is a key determinant of the ability of COX enzymes to function, and this can be influenced by diet [34]. We measured the expression of COX enzymes in ovaries to see if it is influenced by dietary fish oil. The expression of COX-1 protein and mRNA was significantly decreased in ovaries of 100 and 175 mg/kg fish oil-fed hens. The COX and PGE_2_ endpoints are tightly correlated with the incorporation of DHA into the yolks. This was in agreement with the studies that showed the DHA [35] and EPA [4] found in fish oil are effective inhibitors of COX-1. We and others have shown that COX-1 is over-expressed in ovarian cancer [3,6,7]. The regulation of PGE_2_ synthesis by COX-1 in ovarian cancer cell lines [36] has been previously shown. Targeting COX-1 in ovarian cancer is one of the beneficial actions of fish oil which may attenuate and even suppress this disease.

Several studies have shown that OM-3FA enriched diets inhibit COX-2 and prostaglandins in both plasma and experimentally induced tumors [37] but the effect of fish oil on COX-2 expression in ovaries of hens was unknown. Our data indicated that COX-2 expression at the transcriptional level was decreased in ovaries of hens fed 100 and 175 mg/kg of fish oil resulting in decreased COX-2 protein. This was in agreement with a study that reported a significant decrease in COX-2 expression both in the colonic mucosa and in colon tumors of rats fed fish oil [38]. Aronson *et al.* showed that fish oil decreases the COX-2 expression in prostatic tissue of men [39]. The mechanism through which fish oil reduces the expression of COX-2 remains to be completely elucidated but the inhibitory effect of DHA on COX-2 promotor activity, mRNA levels and protein expression in human endothelial cells has been reported [40]. NF-κB is a transcription factor that plays an important role in various inflammatory signaling pathways and controls effectors enzymes such as COX-2 [41]. Previous studies have shown that fish oil supplementation inhibited NF-κB activation [42,43] which in turn leads to down-regulation of COX-2.

Previous findings have demonstrated that fish oil supplementation in human diets decreases production of PGE_2_[44-47], but the effect of fish oil on PGE_2_ concentration in hen ovaries had not been investigated. Our results indicated that there was a statistically significant reduction in concentration of PGE_2_ in ovaries of hens fed 100 and 175 mg/kg fish oil compared to controls. The mechanisms by which the OM-3FAs inhibit PGE_2_ remain to be fully elucidated. Dietary OM-3FAs may modulate substrate pools available to COXs and lipoxygenases (LOX), thereby controlling the downstream eicosanoid formation and subsequent receptor activation [48]. COX enzymes convert OM-6FAs to 2-series PG products such as PGE_2_ whereas the end products of COX enzymes activity on OM-3FAs are 3-series prostaglandins such as PGE_3_. The 3-series PG products are generally less pro-inflammatory than the 2-series products [49-51]. Prostaglandins are potent mediators of intercellular communication, and high concentrations of PGE_2_ are believed to be immunosuppressive of T cell-mediated immunity [52], increase angiogenesis [53], stimulate cell proliferation and inhibit apoptosis in ovarian cancer cell lines [54]. Elevation of PGE_2_ in ovarian cancer has been previously reported [55]. We have shown that at the age when ovarian cancer was detected for the first time in hens, PGE_2_ concentration and COX expression were elevated in ovaries [24] pointing to the important role of COX enzymes and PGE_2_ in the early stages of ovarian carcinogenesis. Recently, we reported that consumption of flaxseed enriched diet, the richest plant source of OM-3FAs, reduced the ovarian cancer severity and incidence in hens which was correlated to decreased concentration of PGE_2_ and expression of COX-2 in ovaries [25,56].

EP4 receptor promotes tumor progression by increasing pro-angiogenic factor and tumor cell invasiveness in ovarian carcinoma cell lines. Spinella *et al.* reported that EP2-EP4 signaling regulates vascular endothelial growth factor production and ovarian carcinoma cell invasion [57]. Our study demonstrated that consumption fish oil does not affect expression of EP4 at the level of mRNA or protein.

COX-2 governs innumerable physiological processes including ovulation. PGE_2_ action on granulosa cells is essential for successful ovulation [58]. There was a concern that reduced expression of COX-2 and concentration of PGE_2_ may lead to decreased ovulation rate. We noted a higher number of ovulations in hens fed 100 mg/kg fish oil at the end of third week compared to first week of the study. This finding was in agreement with Broughton *et al*. who has shown that consumption of fish oil resulted in an elevated ovulation rate in rats [30]. Incorporation of OM3-FA appears to enhance ovulation by reducing excess inflammatory PGE_2_ possibly through reduced AA availability and decreased COX-2 [30].

One of the hallmarks of cancer is the net gain of cancerous cells, either through excessive proliferation or through failure of programmed cell death (apoptosis). We postulated that fish oil reduces the proliferation and increases the apoptosis of hen ovarian cells. Our data indicated an increase in the rate of apoptosis in ovaries of the hens fed the highest fish oil dose. Proliferation was lower in the group of hens fed lower doses of fish oil. Our findings were in agreement with previous studies which have shown that fish oil increases apoptosis [59] and decreases proliferation in rat mammary tumors [60]; fish oil prevents colon carcinogenesis by enhancing apoptosis in the colon [61,62]. Thus measurement of apoptosis vs proliferation provides an index of therapeutic efficacy. By targeting apoptosis and proliferation, fish oil at the optimum concentration reduces the pro-carcinogenic environment potentially resulting in prevention of cancer initiation.

In conclusion, our findings show that dietary intervention with fish oil decreases the expression of COX-1, COX-2 and the concentration of PGE_2_ which likely reduces the carcinogenic microenvironment in ovaries of the laying hens. Fish oil has been reported to be immune-suppressive at high concentrations and immune-stimulatory at lower concentration in chickens [31,63,64]. The reduction in PGE_2_ appears to be primarily due to inhibition of both COX enzymes, and it is likely that substrate modulation of both COX enzymes further contributes to the reduction in PGE_2_ levels. It is generally agreed that an effective way to control cancer is to find better ways of preventing it. Chemopreventive approaches are recommended for women who have a family history of cancer or express other known risk factors for ovarian cancer [65]. To our knowledge the present study provides the first insight into the efficacy of fish oil on the reduction of COX enzyme expression and PGE_2_ concentration in the normal pre-cancerous ovary and further demonstrates the utility of the hen model for ovarian cancer. Our study provides new insight into the potential mechanism of action of fish oil in the reduction of ovarian cancer and will establish the foundation for clinical trials to test the efficacy of dietary intervention with fish oil for the prevention and suppression of ovarian cancer in women.

## Competing interests

The authors declare that they have no competing interests.

## Authors’ contributions

EE and DBH designed the experiment and protocols and wrote the manuscript. EE performed animal care and treatment, sample collection, western blot, PCR, PGE_2_ assay, statistical analysis. CCS conducted the gas chromatography analysis. SRM performed TUNEL and PCNA. NKK participated in animal handling, treatment and tissue collection and analysis. DBH and EE interpreted the data. All authors read and approved the final manuscript.

## References

[B1] Williams CS Mann M DuBois RN The role of cyclooxygenases in inflammation, cancer, and development Oncogene 1999 18 55 7908 7916 10630643 10.1038/sj.onc.1203286

[B2] Gupta RA Tejada LV Tong BJ Das SK Morrow JD Dey SK DuBois RN Cyclooxygenase-1 is overexpressed and promotes angiogenic growth factor production in ovarian cancer Cancer Res 2003 63 5 906 911 12615701

[B3] Daikoku T Wang D Tranguch S Morrow JD Orsulic S DuBois RN Dey SK Cyclooxygenase-1 Is a Potential Target for Prevention and Treatment of Ovarian Epithelial Cancer Cancer Res 2005 65 9 3735 3744 10.1158/0008-5472.CAN-04-3814 15867369 PMC2584020

[B4] Smith WL Cyclooxygenases, peroxide tone and the allure of fish oil Curr Opin Cell Biol 2005 17 2 174 182 10.1016/j.ceb.2005.02.005 15780594

[B5] Denkert C Kobel M Pest S Koch I Berger S Schwabe M Siegert A Reles A Klosterhalfen B Hauptmann S Expression of cyclooxygenase 2 is an independent prognostic factor in human ovarian carcinoma Am J Pathol 2002 160 3 893 903 10.1016/S0002-9440(10)64912-7 11891188 PMC1867167

[B6] Urick ME Johnson PA Cyclooxygenase 1 and 2 mRNA and protein expression in the Gallus domesticus model of ovarian cancer Gynecol Oncol 2006 103 2 673 678 10.1016/j.ygyno.2006.05.012 16797680

[B7] Hales DB Zhuge Y Lagman JA Ansenberger K Mahon C Barua A Luborsky JL Bahr JM Cyclooxygenases expression and distribution in the normal ovary and their role in ovarian cancer in the domestic hen (Gallus domesticus) Endocrine 2008 33 3 235 244 10.1007/s12020-008-9080-z 18498063 PMC4445833

[B8] Reese J Zhao X Ma WG Brown N Maziasz TJ Dey SK Comparative analysis of pharmacologic and/or genetic disruption of cyclooxygenase-1 and cyclooxygenase-2 function in female reproduction in mice Endocrinology 2001 142 7 3198 3206 10.1210/en.142.7.3198 11416042

[B9] Greene ER Huang S Serhan CN Panigrahy D Regulation of inflammation in cancer by eicosanoids Prostaglandins Other Lipid Mediat 2011 96 1–4 27 36 21864702 10.1016/j.prostaglandins.2011.08.004 PMC4051344

[B10] Gupta RA Dubois RN Colorectal cancer prevention and treatment by inhibition of cyclooxygenase-2 Nat Rev Cancer 2001 1 1 11 21 11900248 10.1038/35094017

[B11] Terada N Shimizu Y Kamba T Inoue T Maeno A Kobayashi T Nakamura E Kamoto T Kanaji T Maruyama T Identification of EP4 as a potential target for the treatment of castration-resistant prostate cancer using a novel xenograft model Cancer Res 2010 70 4 1606 1615 10.1158/0008-5472.CAN-09-2984 20145136

[B12] Chell SD Witherden IR Dobson RR Moorghen M Herman AA Qualtrough D Williams AC Paraskeva C Increased EP4 receptor expression in colorectal cancer progression promotes cell growth and anchorage independence Cancer Res 2006 66 6 3106 3113 10.1158/0008-5472.CAN-05-3702 16540660

[B13] Ma X Kundu N Rifat S Walser T Fulton AM Prostaglandin E receptor EP4 antagonism inhibits breast cancer metastasis Cancer Res 2006 66 6 2923 2927 10.1158/0008-5472.CAN-05-4348 16540639

[B14] Ansenberger K Zhuge Y Richards C Barua A Luborsky JL Bahr JM Hales DB Decreased severity of ovarian cancer and increased survival in hens fed a flaxseed enriched diet for one year Gynecol Oncol 2010 117 341 347 10.1016/j.ygyno.2010.01.021 20153884 PMC2849883

[B15] Glade MJ Food, nutrition, and the prevention of cancer: a global perspective. American Institute for Cancer Research/World Cancer Research Fund, American Institute for Cancer Research, 1997 Nutrition 1999 15 6 523 526 10378216 10.1016/s0899-9007(99)00021-0

[B16] Sharma A Belna J Espat J Rodriguez G Cannon VT Hurteau JA Effects of omega-3 fatty acids on components of the transforming growth factor beta-1 pathway: implication for dietary modification and prevention in ovarian cancer Am J Obstet Gynecol 2009 200 5 e511 516 10.1016/j.ajog.2008.12.023 19268879

[B17] Simopoulos AP Omega-3 fatty acids in health and disease and in growth and development Am J Clin Nutr 1991 54 3 438 463 1908631 10.1093/ajcn/54.3.438

[B18] Calviello G Serini S Piccioni E n-3 polyunsaturated fatty acids and the prevention of colorectal cancer: molecular mechanisms involved Curr Med Chem 2007 14 29 3059 3069 10.2174/092986707782793934 18220742

[B19] Ansenberger K Zhuge Y Lagman JA Richards C Barua A Bahr JM Hales DB E-cadherin expression in ovarian cancer in the laying hen, Gallus domesticus, compared to human ovarian cancer Gynecol Oncol 2009 113 3 362 369 10.1016/j.ygyno.2009.02.011 19321195 PMC2838372

[B20] Hales DB Zhuge Y Lagman JAJ Ansenberger K Mahon C Narua A Luborsky J Bahr JM Cyclooxygenases expression and distribution in the normal ovary and their role in ovarian cancer in the domestic hen (Gallus Domesticus) Endocrine 2008 in press 10.1007/s12020-008-9080-z PMC4445833 18498063

[B21] Zhuge Y Lagman JA Ansenberger K Mahon CJ Daikoku T Dey SK Bahr JM Hales DB CYP1B1 expression in ovarian cancer in the laying hen Gallusdomesticus Gynecologic oncology 2009 112 1 171 178 10.1016/j.ygyno.2008.09.026 18973935 PMC2741324

[B22] Rodriguez-Burford C Barnes MN Berry W Partridge EE Grizzle WE Immunohistochemical expression of molecular markers in an avian model: a potential model for preclinical evaluation of agents for ovarian cancer chemoprevention Gynecol Oncol 2001 81 3 373 379 10.1006/gyno.2001.6191 11371125

[B23] Barua A Bitterman P Abramowicz JS Dirks AL Bahr JM Hales DB Bradaric MJ Edassery SL Rotmensch J Luborsky JL Histopathology of ovarian tumors in laying hens: a preclinical model of human ovarian cancer Int J Gynecol Cancer 2009 19 4 531 539 10.1111/IGC.0b013e3181a41613 19509547 PMC2759668

[B24] Eilati E Pan L Bahr JM Hales DB Age dependent increase in prostaglandin pathway coincides with onset of ovarian cancer in laying hens Prostaglandins Leukot Essent Fatty Acids 2012 87 6 177 184 10.1016/j.plefa.2012.09.003 23089186 PMC3592969

[B25] Eilati E Bahr JM Hales DB Long term consumption of flaxseed enriched diet decreased ovarian cancer incidence and prostaglandin E in hens Gynecol Oncol 2013 DOI:10.1016/j.ygyno.2013.05.018 10.1016/j.ygyno.2013.05.018 PMC3824958 23707669

[B26] Trebunova A Vasko L Svedova M Kastel R Tuckova M Mach P The influence of omega-3 polyunsaturated fatty acids feeding on composition of fatty acids in fatty tissues and eggs of laying hens Dtsch Tierarztl Wochenschr 2007 114 7 275 279 17724936

[B27] Bautista-Ortega J Goeger DE Cherian G Egg yolk omega-6 and omega-3 fatty acids modify tissue lipid components, antioxidant status, and ex vivo eicosanoid production in chick cardiac tissue Poult Sci 2009 88 6 1167 1175 10.3382/ps.2009-00027 19439626

[B28] Jump DB The biochemistry of n-3 polyunsaturated fatty acids J Biol Chem 2002 277 11 8755 8758 10.1074/jbc.R100062200 11748246

[B29] Schreiner M Hulan HW Razzazi-Fazeli E Bohm J Iben C Feeding laying hens seal blubber oil: effects on egg yolk incorporation, stereospecific distribution of omega-3 fatty acids, and sensory aspects Poult Sci 2004 83 3 462 473 15049501 10.1093/ps/83.3.462

[B30] Broughton KS Bayes J Culver B High alpha-linolenic acid and fish oil ingestion promotes ovulation to the same extent in rats Nutr Res 2010 30 10 731 738 10.1016/j.nutres.2010.09.005 21056289

[B31] Ebeid T Eid Y Saleh A Abd El-Hamid H Ovarian follicular development, lipid peroxidation, antioxidative status and immune response in laying hens fed fish oil-supplemented diets to produce n-3-enriched eggs Animal 2008 2 1 84 91 22444966 10.1017/S1751731107000882

[B32] Meluzzi A Sirri F Manfreda G Tallarico N Franchini A Effects of dietary vitamin E on the quality of table eggs enriched with n-3 long-chain fatty acids Poult Sci 2000 79 4 539 545 10780651 10.1093/ps/79.4.539

[B33] Simopoulos AP Human requirement for N-3 polyunsaturated fatty acids Poult Sci 2000 79 7 961 970 10901194 10.1093/ps/79.7.961

[B34] Smith WL Nutritionally essential fatty acids and biologically indispensable cyclooxygenases Trends Biochem Sci 2008 33 1 27 37 10.1016/j.tibs.2007.09.013 18155912

[B35] Corey EJ Shih C Cashman JR Docosahexaenoic acid is a strong inhibitor of prostaglandin but not leukotriene biosynthesis Proc Natl Acad Sci USA 1983 80 12 3581 3584 10.1073/pnas.80.12.3581 6304720 PMC394093

[B36] Kino Y Kojima F Kiguchi K Igarashi R Ishizuka B Kawai S Prostaglandin E2 production in ovarian cancer cell lines is regulated by cyclooxygenase-1, not cyclooxygenase-2 Prostaglandins Leukot Essent Fatty Acids 2005 73 2 103 111 10.1016/j.plefa.2005.04.014 15963707

[B37] Spencer L Mann C Metcalfe M Webb M Pollard C Spencer D Berry D Steward W Dennison A The effect of omega-3 FAs on tumour angiogenesis and their therapeutic potential Eur J Cancer 2009 45 12 2077 2086 10.1016/j.ejca.2009.04.026 19493674

[B38] Singh J Hamid R Reddy BS Dietary fat and colon cancer: modulation of cyclooxygenase-2 by types and amount of dietary fat during the postinitiation stage of colon carcinogenesis Cancer Res 1997 57 16 3465 3470 9270014

[B39] Aronson WJ Glaspy JA Reddy ST Reese D Heber D Bagga D Modulation of omega-3/omega-6 polyunsaturated ratios with dietary fish oils in men with prostate cancer Urology 2001 58 2 283 288 10.1016/S0090-4295(01)01116-5 11489728

[B40] Massaro M Habib A Lubrano L Del Turco S Lazzerini G Bourcier T Weksler BB De Caterina R The omega-3 fatty acid docosahexaenoate attenuates endothelial cyclooxygenase-2 induction through both NADP(H) oxidase and PKC epsilon inhibition Proc Natl Acad Sci USA 2006 103 41 15184 15189 10.1073/pnas.0510086103 17018645 PMC1622797

[B41] Ghosh S Karin M Missing pieces in the NF-kappaB puzzle Cell 2002 109 Suppl S81 96 11983155 10.1016/s0092-8674(02)00703-1

[B42] Xi S Cohen D Chen LH Effects of fish oil on cytokines and immune functions of mice with murine AIDS J Lipid Res 1998 39 8 1677 1687 9717729

[B43] Wang TM Chen CJ Lee TS Chao HY Wu WH Hsieh SC Sheu HH Chiang AN Docosahexaenoic acid attenuates VCAM-1 expression and NF-kappaB activation in TNF-alpha-treated human aortic endothelial cells J Nutr Biochem 2011 22 2 187 194 10.1016/j.jnutbio.2010.01.007 20573493

[B44] Endres S Ghorbani R Kelley VE Georgilis K Lonnemann G van der Meer JW Cannon JG Rogers TS Klempner MS Weber PC The effect of dietary supplementation with n-3 polyunsaturated fatty acids on the synthesis of interleukin-1 and tumor necrosis factor by mononuclear cells N Engl J Med 1989 320 5 265 271 10.1056/NEJM198902023200501 2783477

[B45] Caughey GE Mantzioris E Gibson RA Cleland LG James MJ The effect on human tumor necrosis factor alpha and interleukin 1 beta production of diets enriched in n-3 fatty acids from vegetable oil or fish oil Am J Clin Nutr 1996 63 1 116 122 8604658 10.1093/ajcn/63.1.116

[B46] Meydani SN Endres S Woods MM Goldin BR Soo C Morrill-Labrode A Dinarello CA Gorbach SL Oral (n-3) fatty acid supplementation suppresses cytokine production and lymphocyte proliferation: comparison between young and older women J Nutr 1991 121 4 547 555 2007907 10.1093/jn/121.4.547

[B47] Swails WS Kenler AS Driscoll DF DeMichele SJ Babineau TJ Utsunamiya T Chavali S Forse RA Bistrian BR Effect of a fish oil structured lipid-based diet on prostaglandin release from mononuclear cells in cancer patients after surgery JPEN J Parenter Enteral Nutr 1997 21 5 266 274 10.1177/0148607197021005266 9323688

[B48] Moonen HJ Dommels YE van Zwam M van Herwijnen MH Kleinjans JC Alink GM de Kok TM Effects of polyunsaturated fatty acids on prostaglandin synthesis and cyclooxygenase-mediated DNA adduct formation by heterocyclic aromatic amines in human adenocarcinoma colon cells Molecular carcinogenesis 2004 40 3 180 188 10.1002/mc.20032 15224350

[B49] Kobel M Reuss A Bois A Kommoss S Kommoss F Gao D Kalloger SE Huntsman DG Gilks CB The biological and clinical value of p53 expression in pelvic high-grade serous carcinomas J Pathol 2010 222 2 191 198 10.1002/path.2744 20629008

[B50] Wada M DeLong CJ Hong YH Rieke CJ Song I Sidhu RS Yuan C Warnock M Schmaier AH Yokoyama C Enzymes and receptors of prostaglandin pathways with arachidonic acid-derived versus eicosapentaenoic acid-derived substrates and products J Biol Chem 2007 282 31 22254 22266 10.1074/jbc.M703169200 17519235

[B51] Bagga D Wang L Farias-Eisner R Glaspy JA Reddy ST Differential effects of prostaglandin derived from omega-6 and omega-3 polyunsaturated fatty acids on COX-2 expression and IL-6 secretion Proc Natl Acad Sci USA 2003 100 4 1751 1756 10.1073/pnas.0334211100 12578976 PMC149905

[B52] Gebauer SK Psota TL Harris WS Kris-Etherton PM n-3 fatty acid dietary recommendations and food sources to achieve essentiality and cardiovascular benefits Am J Clin Nutr 2006 83 6 Suppl 1526S 1535S 16841863 10.1093/ajcn/83.6.1526S

[B53] Rodriguez-Leyva D Dupasquier CM McCullough R Pierce GN The cardiovascular effects of flaxseed and its omega-3 fatty acid, alpha-linolenic acid Can J Cardiol 2010 26 9 489 496 10.1016/S0828-282X(10)70455-4 21076723 PMC2989356

[B54] Munkarah AR Morris R Baumann P Deppe G Malone J Diamond MP Saed GM Effects of prostaglandin E(2) on proliferation and apoptosis of epithelial ovarian cancer cells J Soc Gynecol Investig 2002 9 3 168 173 10.1016/S1071-5576(02)00141-7 12009392

[B55] Kushlinskii NE Podistov Iu I Laktionov KP Karseladze AI Babkina IV Kerimova GI Prostaglandins E in the primary tumor, metastases, and ascitic fluid of patients with ovarian cancer Biull Eksp Biol Med 1997 123 1 83 86 9213468

[B56] Eilati E Hales K Zhuge Y Ansenberger Fricano K Yu R van Breemen RB Hales DB Flaxseed enriched diet-mediated reduction in ovarian cancer severity is correlated to the reduction of prostaglandin E2 in laying hen ovaries Prostaglandins Leukot Essent Fatty Acids 2013 89 4 179 187 10.1016/j.plefa.2013.08.001 23978451 PMC3811136

[B57] Spinella F Rosano L Di Castro V Natali PG Bagnato A Endothelin-1-induced prostaglandin E2-EP2, EP4 signaling regulates vascular endothelial growth factor production and ovarian carcinoma cell invasion J Biol Chem 2004 279 45 46700 46705 10.1074/jbc.M408584200 15347673

[B58] Duffy DM McGinnis LK Vandevoort CA Christenson LK Mammalian oocytes are targets for prostaglandin E2 (PGE2) action Reprod Biol Endocrinol 2010 8 131 10.1186/1477-7827-8-131 21040553 PMC2988801

[B59] Manna S Chakraborty T Ghosh B Chatterjee M Panda A Srivastava S Rana A Dietary fish oil associated with increased apoptosis and modulated expression of Bax and Bcl-2 during 7,12-dimethylbenz(alpha)anthracene-induced mammary carcinogenesis in rats Prostaglandins Leukot Essent Fatty Acids 2008 79 1–2 5 14 18614344 10.1016/j.plefa.2008.05.005

[B60] Manna S Chakraborty T Damodaran S Samanta K Rana B Chatterjee M Protective role of fish oil (Maxepa) on early events of rat mammary carcinogenesis by modulation of DNA-protein crosslinks, cell proliferation and p53 expression Cancer Cell Int 2007 7 6 10.1186/1475-2867-7-6 17470299 PMC1872018

[B61] Hong MY Bancroft LK Turner ND Davidson LA Murphy ME Carroll RJ Chapkin RS Lupton JR Fish oil decreases oxidative DNA damage by enhancing apoptosis in rat colon Nutr Cancer 2005 52 2 166 175 10.1207/s15327914nc5202_7 16201848

[B62] Hong MY Chapkin RS Davidson LA Turner ND Morris JS Carroll RJ Lupton JR Fish oil enhances targeted apoptosis during colon tumor initiation in part by downregulating Bcl-2 Nutr Cancer 2003 46 1 44 51 10.1207/S15327914NC4601_06 12925303

[B63] Wang YW Field CJ Sim JS Dietary polyunsaturated fatty acids alter lymphocyte subset proportion and proliferation, serum immunoglobulin G concentration, and immune tissue development in chicks Poult Sci 2000 79 12 1741 1748 11194036 10.1093/ps/79.12.1741

[B64] Fritsche KL Cassity NA Huang SC Effect of dietary fat source on antibody production and lymphocyte proliferation in chickens Poult Sci 1991 70 3 611 617 10.3382/ps.0700611 2047352

[B65] Wang D Dubois RN Prostaglandins and cancer Gut 2006 55 1 115 122 10.1136/gut.2004.047100 16118353 PMC1856377

